# Force-Free Control for Direct Teaching of a Surgical Assistant Robot End Effector with Wire-Driven Bidirectional Telescopic Mechanism

**DOI:** 10.3390/s21103498

**Published:** 2021-05-17

**Authors:** Youqiang Zhang, Cheol-Su Jeong, Minhyo Kim, Sangrok Jin

**Affiliations:** 1 School of Mechanical Engineering, Pusan National University, Busan 46241, Korea; zhangyq@pusan.ac.kr (Y.Z.); mhkim1@pusan.ac.kr (M.K.); 2 Research Institute of Mechanical Technology (RIMT), Pusan National University, Busan 46241, Korea; tk0083@nate.com

**Keywords:** surgical assistant robot, force-free control, bidirectional telescopic mechanism, direct teaching, LuGre friction model

## Abstract

This paper shows the design and modeling of an end effector with a bidirectional telescopic mechanism to allow a surgical assistant robot to hold and handle surgical instruments. It also presents a force-free control algorithm for the direct teaching of end effectors. The bidirectional telescopic mechanism can actively transmit force both upwards and downwards by staggering the wires on both sides. In order to estimate and control torque via motor current without a force/torque sensor, the gravity model and friction model of the device are derived through repeated experiments. The LuGre model is applied to the friction model, and the static and dynamic parameters are obtained using a curve fitting function and a genetic algorithm. Direct teaching control is designed using a force-free control algorithm that compensates for the estimated torque from the motor current for gravity and friction, and then converts it into a position control input. Direct teaching operation sensitivity is verified through hand-guiding experiments.

## 1. Introduction

**Figure 1:**
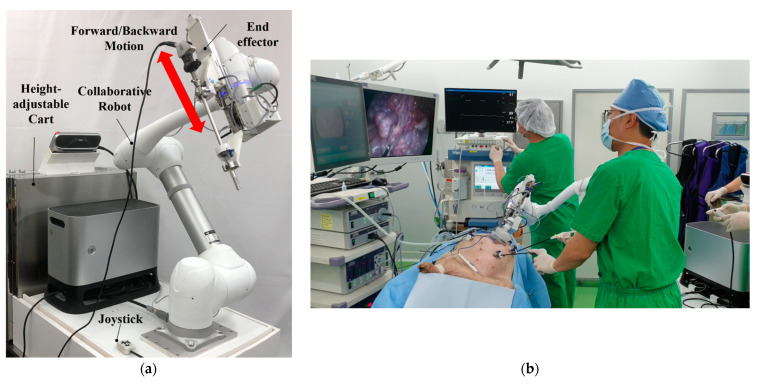
The surgical assistant robot: (**a**) prototype with end effector; (**b**) animal experiments.

In recent years, unlike conventional surgical robots—which perform operations remotely from a console—“surgical assistant robots” that cooperate with surgeons directly at the operating table have received new interest. In laparoscopic surgery, the role of the surgical assistant robot is to move surgical instruments such as endoscopic cameras and forceps in the remote center of motion based on the trocar-inserted port. For efficient motion distribution, the cooperative robot maintains a remote center position and serves to control the orientation of the surgical tool, and the end effector can move back and forth by inserting the surgical tool. In this work, we developed an end effector specialized for surgical support for a commercial collaborative robot, and mounted it as shown in Figure 1a [1]. First, the cooperative robot is manipulated so that the trocar—a port for laparoscopic surgery—can be attached to the trocar mounting of the end effector attached to the end of the cooperative robot. When the trocar is mounted into the end effector, the center of the trocar is defined as the remote center position from that point on. Remote center motion is a cone-shaped movement consisting of a rotation of three degrees of freedom and a translational motion of one degree of freedom. The cooperative robot is responsible for the rotational motion of three degrees of freedom, and the end effector in this study is responsible for the translational motion of one degree of freedom. For example, when an endoscopic camera is equipped with an end effector, the rotational motion of three degrees of freedom changes the direction and slope of the endoscopic camera screen, while the translational motion of one degree of freedom manipulates the zoom distance of the screen. As shown in Figure 1b, assistance in manipulating the endoscopic camera was performed during cholecystectomies and appendectomies on pigs, and the surgeries were successfully completed without errors during the 5-h surgical period.

A telescopic mechanism is used to implement the forward and backward motion of the end effector within a compact structure. Unlike the ball screw guide used in the da Vinci Xi (Intuitive Surgical, Inc., Sunnyvale, CA, USA), when the endoscopic tool is inserted, the structure folds and becomes smaller, reducing the collision with the surgeon on the operating table. Wires are used as the medium by which to connect and drive the telescopic structure in order to minimize the size of the end effector. The general telescopic structure is actively driven in the direction of overcoming gravity, and passively operates using gravity in the direction of gravity. However, a bidirectional telescopic structure must be designed, because surgery requires both the pressing and pulling forces of the tool. In the bidirectional telescopic structure, the wires of the driven slide are connected in the opposite shape [2]. In this study, a two-stage bidirectional telescopic mechanism is designed, and static analysis is performed.

Direct teaching is the most basic and intuitive way to operate a robot that works with surgeons on the operating table. The use of a joystick allows for detailed manipulation, but an approach similar to collaboration with a scrub nurse in standard surgery is direct teaching. Direct teaching means manipulating the motored mechanism with one’s own hands, without using a controller or similar device. This is also known as “manual traction”, “hand-guided control”, or “free mode”. Direct teaching also has a shorter learning curve compared to joysticks. When the surgeon wants to change the direction and position of the endoscopic camera during surgery, the robot operates smoothly according to the surgeon’s intention when the surgeon presses the button on the end effector and applies force. This is akin to dragging the hand of a surgical assistant holding an endoscopic camera to move it to the desired position, and so no training or practice on how to operate is needed. Recently, various studies have been conducted to estimate force/torque and control direct teaching without sensors using a cooperative robot. The external force is estimated from the current value of the motor, and torque control is performed accordingly [3,4], while Zeor Moment Point is also performed at each joint position of the robot [5]. Torque control and impedance control are widely applied in direct teaching [6,7], and observers are also designed to estimate accurate friction models [8,9]. In this study, force-free control based on position control is applied. It is possible to operate in position control using a joystick when performing detailed operations, as needed. Unlike impedance control, this can be applied to any motion according to the force exerted by an external force, without a required trajectory. In addition, in impedance control, an accurate gravity model and friction model must be compensated for; but in force-free control, inaccuracy of the model can be corrected through gain tuning [10,11]. Users may also need to gently manipulate the robot’s end effector, as if holding, inserting, and pulling out surgical tools such as endoscopes. Therefore, force-free control with compensated gravitational and frictional forces is needed. Because a compact structured end effector is equipped with a small control board, it is simplified to force-free control without the need for a large computational control algorithm, such as an observer.

A suitable friction model is also required for the design of a force-free controller. It is important that the friction model account for stiction, the Stribeck effect, and pre-sliding displacement [12]. Wire-driven telescopic structures can be greatly influenced by hysteresis. The LuGre model, which depicts all four aforementioned friction elements while simultaneously having an appropriate computational capacity, is suitable for the mechanism model. The LuGre model is used to control wire-driven surgical robots [13], and is also used for the adaptive control of servo systems [14]. The parameters of the LuGre model are derived through experiments, and reflect the actual system characteristics.

The major contribution of this paper is to control the direct teaching of the end effector with only the measurement of motor current, using the LuGre friction model and force-free control. A bidirectional telescopic mechanism is designed for the surgical assistant end effector, and a control algorithm is applied to the mechanism in order to verify its performance experimentally. This paper is structured as follows: In Section 2, the structure of an end effector with a bidirectional telescopic mechanism is described, and a static model for the load is derived. In Section 3, we derive the gravity and friction models in experiments while designing the force-free control. In particular, the static and dynamic parameters of the friction model—the LuGre model—are obtained experimentally. In Section 4, the performance of the proposed control method is verified experimentally. We conclude in Section 5.

## 2. Bidirectional Telescopic Mechanism

### 2.1. Description of Mechanism

**Figure 2:**
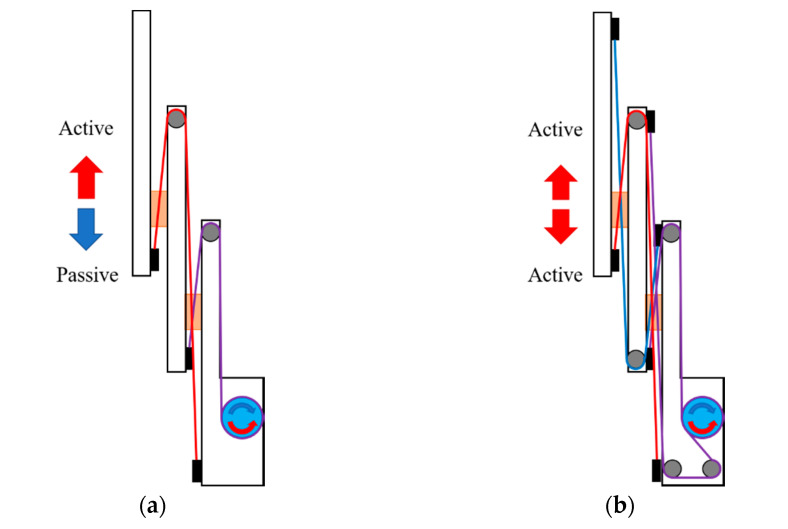
Wire connections comparison: (**a**) one-directional telescopic mechanism; (**b**) bidirectional telescopic mechanism.

**Figure 3:**
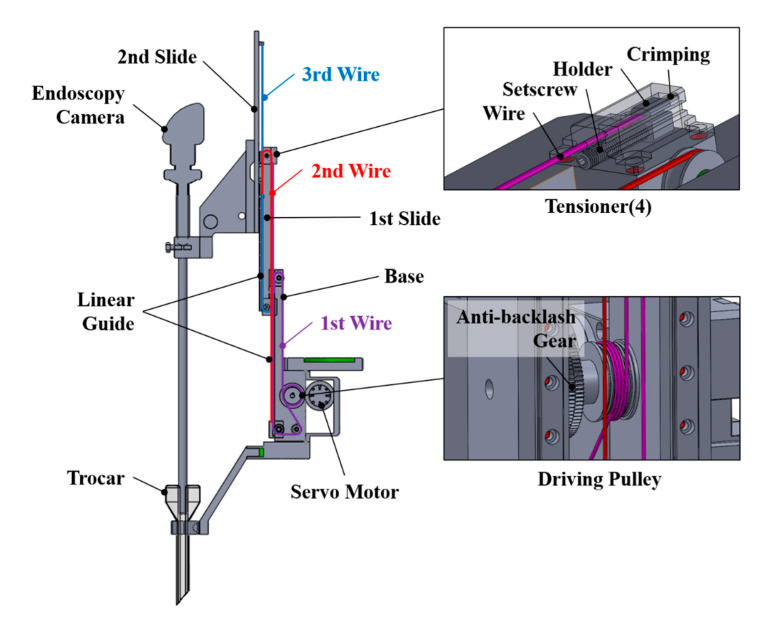
Design of an end effector with a bidirectional telescopic mechanism.

Telescopic mechanisms are used for various purposes because they have a compact structure and can move in a linear motion with a long stroke. Their most significant feature is that they can be folded and unfolded according to the movement distance. In this study, since the ends of the telescopic mechanism must be able to apply force in both directions, a bidirectional telescopic mechanism that crosses and connects wires is designed. A typical telescopic structure has wires connected as shown in Figure 2a. In such a mechanism, the slide goes up when the driving pulley is rotated to apply tension, and then the driving pulley reverses to release tension, causing the slide to go down due to gravity. However, as shown in Figure 2b, a bidirectional telescopic structure that intersects wires and connects them can always maintain tension regardless of the direction of rotation of the driving pulley, and actively generates forces in both ascending and descending motions. The end effector that holds the endoscope camera and moves up and down is configured as shown in Figure 3. There is a driving pulley that drives the wire on the base, and the first wire (purple) is wound around the pulley, which is grooved diagonally and fixed by friction. The driving pulley is rotated by the motor via an anti-backlash gear. Both ends of the wire wound on the pulley pass through the pulley at each end of the first slide, and are fixed to the tensioner by crossing in an “x” shape. The tensioner adjusts the tension by pushing the crimped wire into the holder. The initial tension on each side is applied equally to the other, so that loosening does not occur during driving. Each pulley on the first slide acts as a fixed pulley. The first slide can move up and down according to the direction of the pulley’s rotation, creating movement and bidirectional forces. The second wire (red) and the third wire (blue) cross and connect in an x-shape, so that the movement of the second slide moves in both directions with the first slide. The second and third wires connect the base and the second slide to one another through the pulley of the first slide in the middle. The second and third wires can also be tensioned using tensioners on the base. The pulley on the first slide functions like a moving pulley. The key to the bidirectional telescopic mechanism is that the second and third wires are connected opposite one another. The minimum height of the end effector is 256 mm, and the stroke is 236 mm. The other configuration parameters are shown in Table 1.

### 2.2. Static Modeling

**Figure 4:**
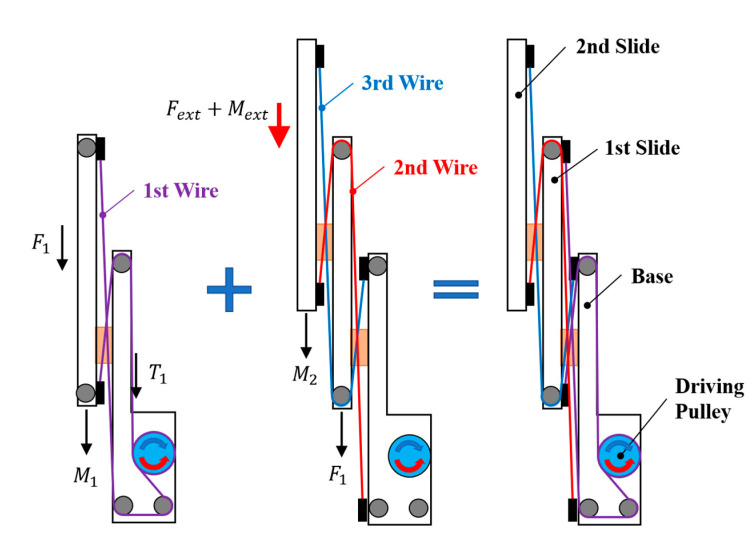
Free body diagram of the bidirectional telescopic mechanism.

The telescopic mechanism has the working principle that the first slide moves when the driving pulley pulls the first wire, while the pulley of the first slide pulls the second slide together with the second or third wire. To derive a static model, the tension model of the first wire and the tension of the second and third wires are divided, as shown in Figure 4. The tension *T*_1_ received by the first wire is determined by the weight *M*_1_ of the first slide and the external force *F*_1_ received by the first slide, as shown in Equation (1). The external force *F*_1_ received by the first slide is determined by the weight *M*_2_ of the second slide and the additional load *M_ext_* and the external force *F_ext_* applied to the second slide, as shown in Equation (2). When the pulley of the first slide acts as a moving pulley, the load it receives is twice the tension of the second and third wires. Therefore, substituting Equation (2) into Equation (1), the tension received by the first wire can be obtained, as in Equation (3), and the required torque is derived by multiplying the radius of the driving pulley, as in Equation (4). The weights *M*_1_, *M*_2_, and *M_ext_* are respectively defined as in Equation (5), while *m*_1_, *m*_2_, *m_ext_* are the mass of the slides and the additional load, *g* is the gravitational acceleration, and *α* and *β* are the tilt angles of the end effector. The tilt angles *α* and *β* are determined by the posture of the end effector. Torque τ_1_ is a control variable. The end effector is mounted at the end of the cooperative robot, which has six degrees of freedom. The tilting angles are factors determined by the joint angles of the cooperative robot, which are determined from the outside, rather than the controlling variable from the end effector’s point of view.

(1) $$T_{1}=M_{1}+F_{1},$$

(2) $$F_{1}=2\left(M_{2}+M_{ext}+F_{ext}\right),$$

(3) $$T_{1}=M_{1}+2\left(M_{2}+M_{ext}+F_{ext}\right),$$

(4) $$\tau_{1}=T_{1}\cdot r,$$

(5) $$M_{1}=m_{1}g\cos\alpha \cos\beta ,\;\;M_{2}=m_{2}g\cos\alpha \cos\beta ,\;\;M_{ext}=m_{ext}g\cos\alpha \cos\beta .$$

## 3. Force-Free Control with Compensation

### 3.1. Gravity Compensation

**Figure 5:**
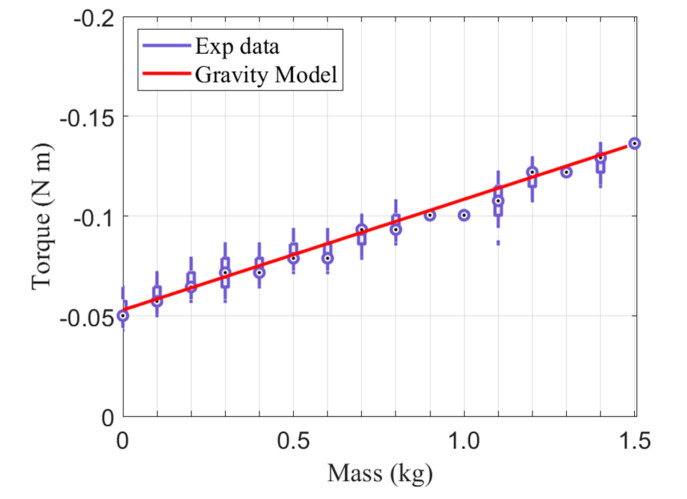
Torque due to the gravitational force of the additional mass.

When teaching directly, gravity and friction must be compensated for, in order to separate the external and internal forces. Gravity was compensated for using the static model obtained in Section 2.2. By multiplying the torque of the drive pulley due to the additional mass by the gear ratio of the anti-backlash gear and the motor reducer, the torque acting on the motor as a result of gravity was calculated, as shown in Equation (6). The experiment of measuring the current by increasing the mass on the second slide of the end effector to 1.5 kg in 0.1 kg increments was repeated. The force required for manual manipulation was 5 N, and the endoscope weighed 0.4 kg. Thus, a gravitational model was derived using up to 1.5 kg of weights. In this study, since torque is estimated by the current value of the motor without a force/torque sensor, the measured current was multiplied by the torque coefficient *K*, as shown in Equation (7), and the result of the additional mass experiment was converted into torque. As shown in Figure 5, we observed that the torque estimated by the experiment fit well with the gravitational torque based on the static model, with an average error of 0.96%. The parameters of the gravity model are shown in Table 2.

(6) $$\tau_{g}=\tau_{1}\cdot N_{1}\cdot N_{2},$$

(7) $$\tau_{est}=K\cdot I.$$

### 3.2. Friction Compensation

Since friction is a dynamic characteristic, more careful modeling is required. The LuGre model applied in this study has the advantage of not having a large computational load, while describing the friction characteristics well; thus, it is used for modeling various motor drive systems [15,16]. The LuGre model is expressed as Equation (8). *z* is an internal variable of the friction model, which represents the average deflection of the bristle. *ω* is the rotational speed of the motor. There are six required parameters: bristle stiffness, bristle damping coefficient, viscous damping coefficient, Coulomb friction level, static friction level, and Stribeck velocity. The LuGre model is divided into static analysis and dynamic analysis, and parameters are obtained through experiments [17,18]. Motor current is measured as rotational speed *ω* is controlled. Torque *τ_d_* is estimated from the measured current through Equation (7).

(8) $$\tau_{d}=\sigma_{0}z+\sigma_{1}\frac{dz}{dt}+\sigma_{2}\omega ,\;\;\;\;\;\;\;\frac{dz}{dt}=\omega -\frac{\sigma_{0}\left|\omega \right|}{\tau_{c}+\left(\tau_{s}-\tau_{c}\right)e^{-{\left(\frac{\omega }{\omega_{s}}\right)}^{2}}}z.$$

#### 3.2.1. Estimating Static Parameters

**Figure 6:**
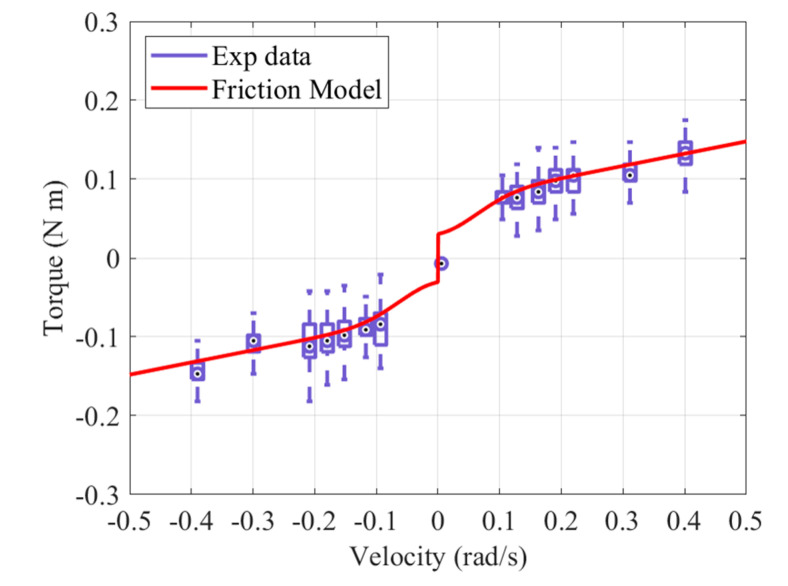
Stribeck curve for the static friction model.

There are four static parameters: Coulomb friction level, static friction level, Stribeck velocity, and viscous damping coefficient. The static parameters can be obtained from the static friction model, as shown in Equation (9). The motor speed varies in 7 values from 0.098 rad/s to 0.39 rad/s, and the steady-state current is measured, while torque is estimated when rotating in a forward/backward direction. The experiment was conducted with the end effector lying on its side, so as not to be affected by gravity. Using the curve fitting algorithm of MATLAB (MathWorks, Natick, MA, USA) matches the experimental results with the static friction model, as shown in Figure 6. As a result of the fitting, the adjusted R square is 0.9965. The derived static parameters are shown in Table 3.

(9) $$\tau_{d,ss}=\left\{\tau_{c}+\left(\tau_{s}-\tau_{c}\right)e^{-{\left(\frac{\omega }{\omega_{s}}\right)}^{2}}\right\}\mathrm{sgn}(\omega )+\sigma_{2}\omega .$$

#### 3.2.2. Estimating Dynamic Parameters

**Figure 7:**
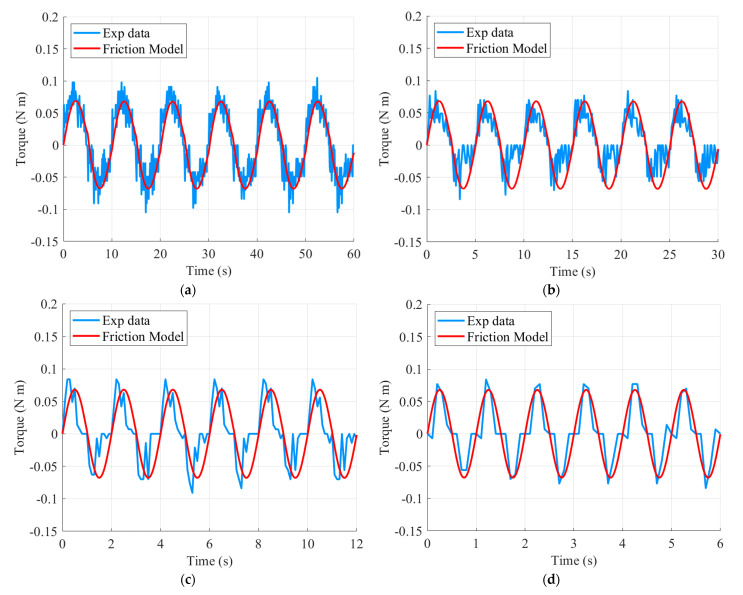
Sinusoidal motion for the dynamic friction model: (**a**) 0.1 Hz; (**b**) 0.2 Hz; (**c**) 0.5 Hz; and (**d**) 1.0 Hz.

There are two dynamic parameters: Bristle stiffness and bristle damping coefficient. The dynamic parameters can be obtained by comparing the results of Equation (8), in which the static parameter values are substituted, with the experimental results. The velocity control input frequency of the motor is given in sine waves of 0.1 Hz, 0.2 Hz, 0.5 Hz, and 1.0 Hz, while the torque is estimated from the obtained current value. The motor has a no-load speed of 60 RPM, and the frequency of the speed of motion is derived from the results of the direct teaching experiment during the surgical simulation. The objective function is defined as in Equation (10), and the dynamic parameters are derived by fitting the model and the experimental results, as shown in Figure 7, using a genetic algorithm. ${\tilde{\tau }}_{d}$ is the estimated friction torque from the experimental data. The derived dynamic parameters are shown in Table 4.

(10) $$\min{\sum \int \left\{\tau_{d}\left(\sigma_{0},\sigma_{1},t\right)-{\tilde{\tau }}_{d}\left(\sigma_{0},\sigma_{1},t\right)\right\}}^{2}dt$$

### 3.3. Design of Force-Free Control

**Figure 8:**
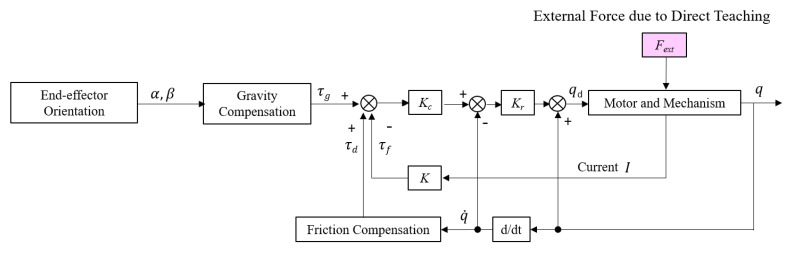
Block diagram of the force-free control.

Force-free control is designed as in Equation (11) [10], and is based on position control, as shown in Figure 8. A new target position is derived by adding the calculated degree of positional change to the current motor position. The degree of positional change is calculated by considering the torque caused by the external force as a result of compensating for gravity and friction. As a result, the external force from the direct teaching becomes the position control input of the end effector. In this work, we focus on obtaining sensitivity so that the slides follow smoothly when users hold the instrument by hand. It is important to control the instrument so that it can easily move and stop immediately according to the user’s intention, even with a force of about 5 N. The direct teaching sensitivity can be tuned by adjusting the control gain *K_c_*, and the gain *K_r_*, which converts the torque into position command. First, *K_r_* was adjusted to set the desired speed, and then manipulation sensitivity was adjusted through *K_c_*. Vibration can occur if *K_r_* is set too high to create a control input faster than the motor’s capacity. Gain was adjusted according to the values shown in Table 5, and the experiment was performed.

(11) $$q_{d}=K_{r}\left\{K_{c}\left(-\tau_{f}+\tau_{d}+\tau_{g}\right)-\dot{q}\right\}+q$$

## 4. Experiment of Direct Teaching Performance

### 4.1. Description of Experimental Apparatus

**Figure 9:**
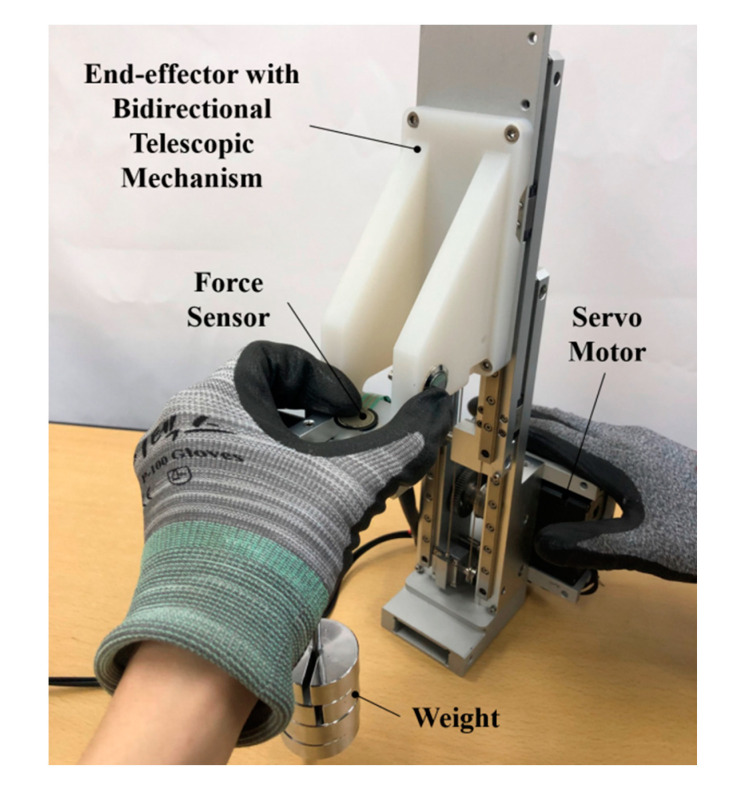
Experimental apparatus.

The prototype end effector was manufactured with a weight of 2.7 kg, a width of 58 mm, a minimum height of 256 mm, and a maximum height of 492 mm. Stainless steel (SUS304) wire with a diameter of 0.81 mm and a cross-sectional structure of 7 × 7 was used. The servo motor (DYNAMIXEL XH540-V150-R, ROBOTIS Co., Ltd., Seoul, Korea) was controlled by the motor drive (DYNAMIXEL Shield, ROBOTIS Co., Ltd., Seoul, Korea) and control board (ARDUINO UNO Rev3). As it was a compact end effector, it was important to be able to control it with only a small control board. When a force was applied to the end effector while pressing the teach button, the slide would be moved up and down by the control algorithm. A force-sensing resistor (FSR SEN0047, Interlink Electronics, Inc., Los Angeles, CA, USA) was installed in order to evaluate the operational sensitivity. The FSR (force-sensing resistor) was chosen to fit the force range of 1–100 N required for the experiment. In addition, the FSR was calibrated using the weights for the balances used in the gravitational model experiment. The experiment was carried out by attaching a weight of 0.4 kg, equivalent to the weight of the endoscopic camera, to the end effector, as shown in Figure 9. If the basic gravity compensation model is set based on the endoscopic camera, the slide slowly rises up without applying force when a button is pressed without a camera, making it easy to mount the camera.

### 4.2. Hand Guiding Experiment

**Figure 10:**
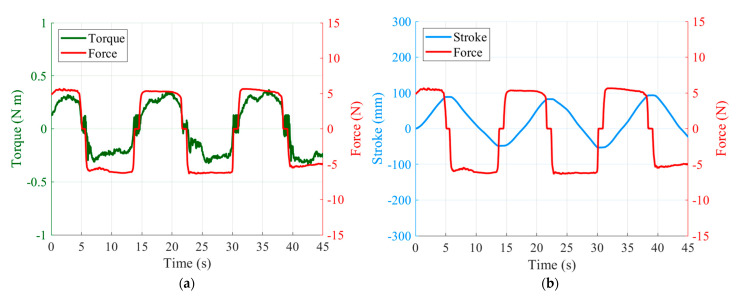
Experimental results of hand guiding: (**a**) estimated torque and force plot; (**b**) stroke and force plot.

**Figure 11:**
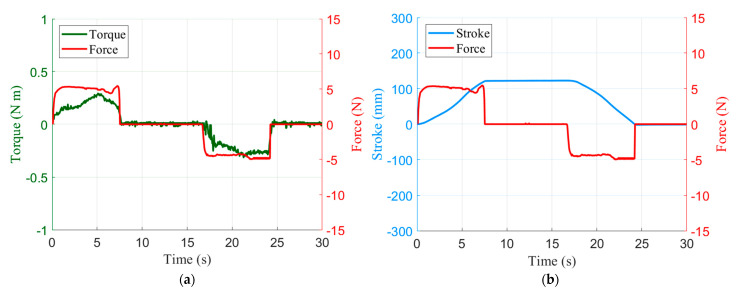
Experimental results of stationary motion: (**a**) estimated torque and force plot; (**b**) stroke and force plot.

When the user grasps the part with the FSR and applies a force upwards or downwards, the motion and the force of the slide can be observed, and the operational sensitivity can be evaluated. As shown in Figure 10a, when an external force is applied, the torque can be estimated from the current. As shown in Figure 10b, the position of the slide can be smoothly controlled according to the external force. When an average force of 5.56 N is applied, the slide moves up and down at a speed of 19.20 mm/s. In addition, if the user applies an external force and then releases the hand from the operating point, the slide stops and remains stable, as shown in Figure 11. When both upward and downward forces are applied and stopped, there is no overshoot or oscillation, and the motor completely stops within a 48 ms delay after the force is removed. This is evidence that gravity and friction are being properly compensated for. The operational sensitivity usually shows various results according to the subjective evaluation, but here it was adjudged to be sufficient to handle the surgical tools. Surgeons, who comprise the market for this robot, require the lightest operational sensitivity possible. When tuning with a lighter sensitivity, fine vibrations occurred, and so further improvement is needed.

## 5. Conclusions

This study deals with the bidirectional telescopic mechanism and direct teaching control of an end effector mounted on a surgical assistant robot. The bidirectional telescopic mechanism allows the surgical instrument to generate active forces both upwards and downwards, by connecting the wires crosswise. It also reduces the risk of collision with the surgeon on the operating table during surgery, because the slides fold according to the stroke. When the surgeon needs to control the end effector directly without manipulating the joystick, it can be guided by hand. The force applied to the end effector is estimated from the motor current, without force/torque sensors, and the direct teaching motion is controlled using force-free control. In order to build a force-free controller, a gravity model and a friction model were experimentally derived. The friction model is derived from the static and dynamic parameters based on the LuGre model. As demonstrated by the hand-guiding experiments, the slide moves up and down at a speed of 19.20 mm/s when an average force of 5.56 N is applied. When no force is applied, gravity and friction are successfully compensated for, allowing the slide to stop and maintain a stable position. Follow-up studies are planned in order to achieve a lighter operational sensitivity.

## Figures and Tables

**Table 1:** Configuration parameters of the end effector.

Parameters	Value	
Height	min	max
	256 mm	492 mm
Width	57 mm	
Whole body weight	2.09 kg	

**Table 2:** Parameters for the gravity model.

Parameters	Description	Value
*m*_1_	Mass of the first slide	0.176 kg
*m*_2_	Mass of the second slide	0.427 kg
*K*	Torque coefficient	2.67 N·m/A
*N*_1_	Gear ratio of the anti-backlash gears	6:7
*N*_2_	Gear ratio of the motor reducer	1:152.3

**Table 3:** Static parameters of the LuGre model.

Parameters	Description	Value
*τ_c_*	Coulomb friction level	0.070 N·m
*τ_s_*	Static friction level	0.030 N·m
*σ*_2_	Viscous damping coefficient	0.155 N m·s/rad
*ω*_2_	Stribeck velocity	0.090 rad^−1^

**Table 4:** Dynamic parameters of the LuGre model.

Parameters	Description	Value
*σ*_0_	Bristle stiffness	0.051 N·m/rad
*σ*_1_	Bristle damping coefficient	0.297 N·m·s/rad

**Table 5:** Gain parameters of the force-free control.

Parameters	Description	Value
*Kr*	Command conversion gain	2.05
*Kc*	Control loop gain	1.68
*K*	Torque coefficient	2.67 ^1^

^1^ This value is determined in Table 1.

## Data Availability

The data presented in this study are available on request from the corresponding author.

## References

[1] Kim M., Zhang Y., Jin S. (2021). Control Strategy for Direct Teaching of Non-Mechanical Remote Center Motion of Surgical Assistant Robot with Force/Torque Sensor. Appl. Sci..

[2] Lee D., Chang D., Shin Y.I., Son D., Kim T.-W., Lee K.-Y., Kim J. (2011). Design and application of a wire-driven bidirectional telescopic mechanism for workspace expansion with a focus on shipbuilding tasks. Adv. Robot..

[3] Du J., Yuan J., Han Z., Qian Y. Current-Based Direct Teaching for Industrial Manipulator. Proceedings of the IEEE International Conference on Robotics and Biomimetics (ROBIO).

[4] Yen S.H., Tang P.C., Lin Y.C., Lin C.Y. (2019). Development of a Virtual Force Sensor for a Low-Cost Collaborative Robot and Applications to Safety Control. Sensors.

[5] Chen S., Luo M., Jiang G., Abdelaziz O. (2018). Collaborative robot zero moment control for direct teaching based on self-measured gravity and friction. Int. J. Adv. Robot. Syst..

[6] Pérez-Ubeda R., Zotovic-Stanisic R., Gutiérrez S.C. (2020). Force Control Improvement in Collaborative Robots through Theory Analysis and Experimental Endorsement. Appl. Sci..

[7] Lee S.D., Ahn K.H., Song J.B. Torque control based sensorless hand guiding for direct robot teaching. Proceedings of the IEEE/RSJ International Conference on Intelligent Robots and Systems (IROS).

[8] Kallu K.D., Abbasi S.J., Khan H., Wang J., Lee M.C. (2019). Implementation of a TSMCSPO controller on a 3-dof hydraulic manipulator for position tracking and sensor-less force estimation. IEEE Access.

[9] Nakamura H., Ohishi K., Yokokura Y., Kamiya N., Miyazaki T., Tsukamoto A. (2018). Force sensorless fine force control based on notch-type friction-free disturbance observers. IEEJ J. Ind. Appl..

[10] Goto S. (December 2006). Forcefree Control for Flexible Motion of Industrial Articulated Robot Arms. Industrial Robotics: Theory, Modelling and Control.

[11] Kushida D., Nakamura M., Goto S., Kyura N. (2001). Human direct teaching of industrial articulated robot arms based on force-free control. Artif. Life Robot..

[12] Pennestrì E., Rossi V., Salvini P., Valentini P.P. (2016). Review and comparison of dry friction force models. Nonlinear Dynammics.

[13] Do T.N., Tjahjowidodo T., Lau M.W.S., Phee S. (2015). Nonlinear friction modelling and compensation control of hysteresis phenomena for a pair of tendon-sheath actuated surgical robots. Mech. Syst. Signal. Process..

[14] Wang X., Wang S. (2012). High performance adaptive control of mechanical servo system with LuGre friction model: Identification and compensation. J. Dyn. Syst. Meas. Control..

[15] Freidovich L., Robertsson A., Shiriaev A., Johansson R. (2010). LuGre-Model-Based Friction Compensation. IEEE Trans. Control. Syst. Technol..

[16] Ishikawa J., Tei S., Hoshino D., Izutsu M., Kamamichi N. Friction compensation based on the LuGre friction model. Proceedings of the SICE Annual Conference 2010.

[17] Piatkowski T. (2014). Dahl and LuGre dynamic friction models. Anal. Sel. Prop. Mech. Mach. Theory.

[18] Wen Y., Chu M., Sun H. (2015). Friction parameters identification and compensation of LuGre model base on genetic algorithms. 2015 International Symposium on Computers & Informatics.

